# Reactive & Efficient: Organic Azides as Cross-Linkers in Material Sciences

**DOI:** 10.3390/molecules25041009

**Published:** 2020-02-24

**Authors:** Marvin Schock, Stefan Bräse

**Affiliations:** 1 Institute of Organic Chemistry, Karlsruhe Institute of Technology (KIT), Fritz-Haber-Weg 6, 76131 Karlsruhe, Germany; marvinschock@googlemail.com; 2 Institute of Biological and Chemical Systems—FMS (IBCS-FMS), Karlsruhe Institute of Technology (KIT), Hermann-von-Helmholtz-Platz 1, 76344 Eggenstein-Leopoldshafen, Germany; 3 3DMM2O—Cluster of Excellence (EXC-2082/1–390761711), Karlsruhe Institute of Technology (KIT), Fritz-Haber-Weg 6, 76131 Karlsruhe, Germany

**Keywords:** azides, photochemistry, polymers, thermosets, nitrenes

## Abstract

The exceptional reactivity of the azide group makes organic azides a highly versatile family of compounds in chemistry and the material sciences. One of the most prominent reactions employing organic azides is the regioselective copper(I)-catalyzed Huisgen 1,3-dipolar cycloaddition with alkynes yielding 1,2,3-triazoles. Other named reactions include the Staudinger reduction, the aza-Wittig reaction, and the Curtius rearrangement. The popularity of organic azides in material sciences is mostly based on their propensity to release nitrogen by thermal activation or photolysis. On the one hand, this scission reaction is accompanied with a considerable output of energy, making them interesting as highly energetic materials. On the other hand, it produces highly reactive nitrenes that show extraordinary efficiency in polymer crosslinking, a process used to alter the physical properties of polymers and to boost efficiencies of polymer-based devices such as membrane fuel cells, organic solar cells (OSCs), light-emitting diodes (LEDs), and organic field-effect transistors (OFETs). Thermosets are also suitable application areas. In most cases, organic azides with multiple azide functions are employed which can either be small molecules or oligo- and polymers. This review focuses on nitrene-based applications of multivalent organic azides in the material and life sciences.

## 1. Introduction

The characteristics of being explosive, malodorous, and highly volatile are usually adverse properties of a compound in the lab. Therefore, after the preparation of the first organic azide, phenyl azide, by Peter Grieß in 1864, interest in this new family of compounds was low for many years [1]. It was not until the 1950s and 1960s that safer derivatives were prepared which allowed the discovery of reactivities that would soon lead to an impressive library of powerful named reactions. At the same time, chemists at Kodak Inc. developed the first photoresist based on organic bisazides, laying an important cornerstone both for the semiconductor industry and organic azides [2]. After this commercial success, organic azides enjoyed increasing popularity as polymer crosslinking agents. The crosslinking process can be initiated by thermal activation or photolysis of the crosslinking agent, and nitrogen is released under the formation of nitrenes. These highly reactive species can form covalent bonds with both saturated and unsaturated hydrocarbon chains of polymers. The material’s mechanical properties, like solubility and hardness, are changed [3,4,5,6,7]. In recent years, this process has also proven to be a powerful strategy to boost efficiencies of polymer-based devices such as membrane fuel cells, organic solar cells (OSCs), light-emitting diodes (LEDs), and organic field-effect transistors (OFETs). For example, the morphology of the donor and acceptor material in bulk hetero junction organic solar cells was successfully locked and the long-time stability of the device increased in this way [8]. Due their superior crosslinking efficiency and mild activation methods devoid of the addition of initiators, organic azides have become the compounds of choice in these applications.

During many decades, the explosive side of organic azides has never been used in commercial applications. While heavy-metal azides have served as detonators in explosives technology, organic azides as both potent and safe explosives are as yet unknown. As nitrogen-rich components, organic azides are prone to violent decomposition at elevated temperature and sensitive to impact or light. Nevertheless, stable derivatives exist and can be designed obeying certain rules of structural design. In recent years, azidated polymers as insensitive energetic binders and plasticizers in propellants have come into focus. They are used to boost the performance of explosive blends without sacrificing safety by taking advantage of the energy delivered during the scission reaction of the azido group [9]. Azido binders and plasticizers are considered as green materials, because combustion produces only molecular nitrogen and other non-toxic decomposition products.

### 1.1. General Comments for Azides

It should be noted that azides and in particular polyazides are (sometimes) prone to violent decomposition at elevated temperatures (over 100 °C, depending on the structure). However, they are stable at room temperature and in the absence of light. For safety precautions, please see [10,11,12]:

### 1.2. Synthesis of Azides, General Considerations

For the synthesis of (poly)azides, many suitable protocols have been employed [10,11]:

- Alkyl azides (e.g., 1,6-diazidohexane, see below):
  ◦ Nucleophilic substitution reactions
  ◦ Addition of hydrazoic acid to double bonds
- Aryl azides:
  ◦ Reaction of diazonium salts with azide ions
- Sulfonyl azides (e.g., 4,4-oxydibenzenesulphonylazide (SDO)):
  ◦ Reaction of sulfonyl halides with inorganic azides
  ◦ Reaction of sulfonyl hydrazides with nitric acid
- Carbonyl azides:
  ◦ Reaction of reactive carbonyl compounds with azides

### 1.3. Reaction of Azides—General Considerations

For the reaction of (poly)azides, many suitable reaction have been employed [10,11]:

- Thermal or photolytic generation and further reaction of nitrenes
- Cycloadditions
- Reaction with nucleophiles
  ◦ Staudinger reaction
- Rearrangements

It should be noted at this point that the (quite different) photochemistries of aryl, acyl, and alkyl azides have been investigated in depth [13]. In particular the groups of Platz [14,15,16,17,18] and Gritsan [19,20,21,22,23,24] contributed with many examples, reactions, and kinetics.

## 2. Azido-Based High Energy Materials

Solid propellants and explosives consist of a mixture of a solid fuel or explosive, binder, plasticizer, and oxidizer [25]. Polymeric binders are used to bind together the high energetic material in order to reduce its sensitivity and to enable further mechanical processing. Plasticizers are added to enhance physical properties like the plasticity and viscosity of the binder polymer by decreasing the attraction between polymer chains. Ideally, they should have low glass transition temperatures and high decomposition temperatures. For high performance of the blend, the whole system must have a favorable heat of formation and oxygen balance. Therefore, energetic plasticizers and binders are preferred over inert ones. At the same time, the high stability and low sensitivity of the material are demanded. Moreover, combustion should not produce hazardous gases. As a nitrogen-rich class of compounds, organic azides usually have positive heats of formation, high densities, and high burning rates. During scission of the azide bond, elemental nitrogen and considerable amounts of energy in the range of 90 kcal per azide unit are released [9]. Azido-based binders and plasticizers possess favorable material properties such as low glass transition temperatures and good thermal and hydrolytic stability, as well as low impact sensitivity. Moreover, they are considered green compounds as their combustion produce only nitrogen and other non-toxic decomposition products. In the next two chapters, organic azides as binders and plasticizers in advanced propellants and explosives are presented.

### 2.1. Azido Binders

**Scheme 1:**
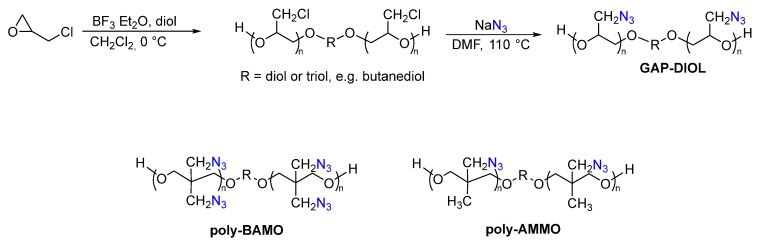
Synthesis of glycidyl azide polymer (**GAP**, top) and structures of poly(3-azidomethyl-3-methyloxetane) and poly(3,3–bis(azidomethyl)oxetane), also known as **poly-AMMO** and and poly-**BAMO** (bottom), respectively.

Several azido polymers have been investigated as energetic binders, such as glycidyl azide polymers (**GAP**) and energetic thermoplastic elastomers (**ETPE**s) such as poly(3-azidomethyl-3-methyloxetane) (**poly-AMMO**) and poly(3,3–bis(azidomethyl)oxetane) (**poly-BAMO**), as well as their copolymers [26]. A common synthesis route for **GAP** is shown in Scheme 1. **Poly-AMMO** and **poly-BAMO** can similarly be obtained from the corresponding oxetanes via ring-opening polymerization and subsequent azidation. So far, only **GAP** has established as a commercially used energetic binder and plasticizer system. Major advantages are its liquid aggregation state at processing temperature, relatively high density, and positive heat of formation, while high glass transition temperature (T_g_ = −43 °C) and poor mechanical characteristics at low temperatures are main demerits. The homopolymer of the most prominent azido oxetane, **BAMO**, has the highest energy output (∆_f_H^0^ = 2.16 kJ g^−1^) of the listed energetic azide polymers, but it is solid because of its symmetric structure and therefore only copolymers can be used as binders. **BAMO** copolymerized with non-energetic monomers like tetrahydrofuran (**THF**) shows good processability and mechanical properties at the expense of lower energy level and thermoplastic character. The copolymer of **BAMO** and azido oxetane **AMMO**, **p(BAMO-AMMO)**, has very attractive features for use as **ETPEs** in extruded propellants and explosives because of its high ∆_f_H^0^ of up to 3.44 kJ g^−1^ although its low temperature properties are not optimal [27].

**Scheme 2:**
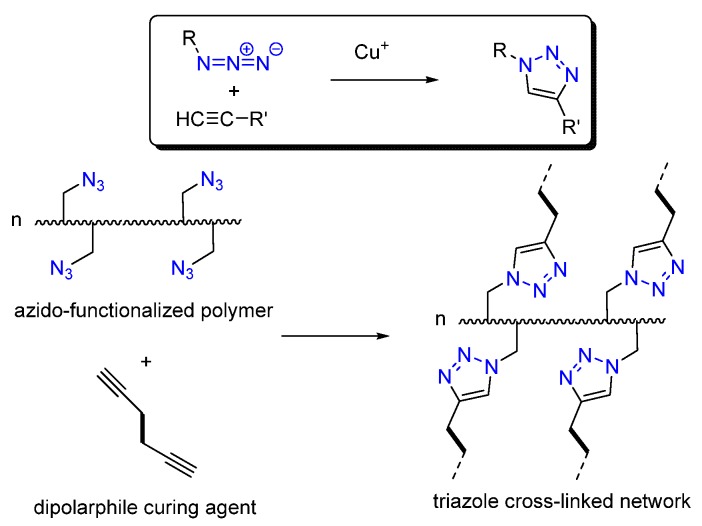
Mechanism of crosslinking of an azido-functionalized polymer with a dipolarophile via 1,3-cycloaddition.

Star azide copolymers with hyperbranched polyether cores and short linear polymeric **BAMO** arms have been prepared by Zhang et al. In contrast to **BAMO** homopolymer, these structures had significantly lower crystallinity and thus greatly improved processability and better safety properties, while maintaining a good energy level of up to 1.37 kJ g^−1^ [28]. To enhance the mechanical properties of the propellant after blending, binders are usually subjected to a process called curing, in which the polymer chains are crosslinked. Isocyanates are traditionally used to cure popular non-energetic polymeric binder hydroxy-terminated polybutadiene (**HTPB**) under the formation of polyurethane networks, and commercial **GAP** is hydroxy terminated for this reason. However, due to their sensitivity to moisture, side reactions with nitrate ester energetic plasticizers, and compatibility problems with chlorine free oxidizer ammonium dinitramide (**ADN**), isocyanate free curing systems have been demanded. Triazole crosslinking between azides and alkynes via 1,3-dipolar cycloaddition was tested successfully [29] (Scheme 2). For example, **GAP**-based triazole networks were prepared using bispropargyl succinate and 1,4-bis(1-hydroxypropargyl)benzene.

While these triazole networks did not show good mechanical properties, a dual curing system employing isocyanate and a small amount of alkyne produced networks with mechanical properties superior to both triazole-only and urethane-only crosslinked networks [30]. Benefits of azido crosslinking include better stability and enhanced burn rate of the crosslinked polymers. For more examples of azido polymers as energetic binders the reader is referred to the literature [26,31,32].

### 2.2. Azido Plasticizers

**Figure 1:**
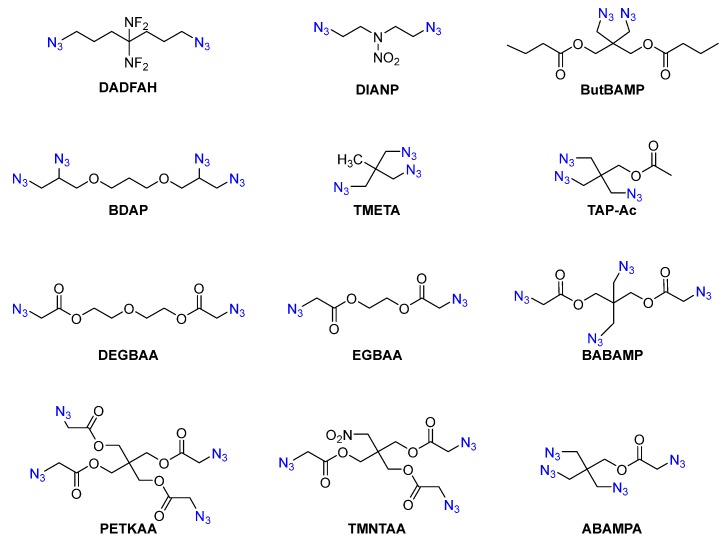
Lines 1 and 2: Azido energetic plasticizers for gun and rocket propellants with different functional groups. Lines 3 and 4: Selected structures of azido acetate esters. From left to right: 1,7-diazido-*N*,*N*,*N*′,*N*′-tetrafluoro-4,4-heptanediamine (**DADFAH**), 1,5-diazido-3-nitrazapentane (**DIANP**), 2,2-bis(azidomethyl)propane-1,3-diyl dibutyrate (**ButBAMP**), bis(2,3-diazidopropylene glycol) (**BDAP)**, 1,1,1-tris(azidomethyl)ethane (**TMETA**). Triazido pentaerythrite acetate (**TAP**-**Ac**), 1,3-bis (azido acetoxy)-2,2-bis(azidomethyl) propane (**BABAMP**), pentaerythritol tetrakis(azidoacetate) (**PETKAA**), trimethylol nitromethane tris(azidoacetate) (**TMNTAA**), 3-azido-2,2-bis(azidomethyl)propyl 2-azidoacetate **(ABAMPA)**.

A recent trend is to use energetic plasticizers (**EP**s) in combination with energetic binders to further increase the performance of the high energetic material. Azido **EP**s have good potential because they show better compatibility with azido binders than commonly used nitrate ester plasticizers due to their structural similarity. Selected structures of azido **EP**s with different functional groups are depicted in Figure 1. A large family of azido EPs are azido acetate esters. In comparison to non-energetic plasticizers, they often exhibit better ballistic, mechanical, and thermal properties [33,34]. They possess high chemical and thermal stability coupled with low sensitivity and glass transition temperatures, although their level of nitrogen content, oxygen balance, density, and heat of formation is not optimal. A large number of azido acetate esters have been prepared, including diethylene glycol bis(azidoacetate) (**DEGBAA**), ethylene glycol bis(azidoacetate) (**EGBAA**), trimethylol nitromethane tris(azidoacetate) (**TMNTAA**), pentaerythritol tetrakis(azidoacetate) (**PETKAA**), and 1,3-bis (azido acetoxy)-2,2-bis(azidomethyl) propane (**BABAMP)** (see Figure 1) [35]. The most recently reported azido ester, 3-azido-2,2-bis(azidomethyl)propyl 2-azidoacetate (**ABAMPA**) has a nitrogen content of 57%, a density of 1.326 g/cm^3^, and a heat of formation $\Delta H_{f}$ of 1725 kJ mol^−1^, revealing better performance than most of the already known azido esters and good compatibility with **GAP** [36]. For an extensive overview on azido EPs, see reference [37]. A method for predicting the glass transition temperature (T_G_) of azido-esters through molecular structures was proposed by Zohari et al. using a training set of 21 compounds [38]. They reported paradoxical behavior regarding the effect of the azide group on T_G_, but their method gave good predictions for a test set and could be of help in designing novel azido-ester plasticizers. A severe problem of small molecule EPs is the migration and exudation from the composite matrix, which deteriorates the mechanical properties and energy level of propellant and thereby reduces their safety. Energetic polymers as plasticizers possess excellent migration resistance due to the inverse correlation between molecular weight and plasticizer mobility. However, linear polymers get too viscous with increasing weight. The highly branched three-dimensional structure of hyperbranched polymers circumvents this issue. Zhang et al. prepared an azido-terminated multi-arm copolymer (**POGA**) with hyperbranched polyether core and linear azido-terminated glycidyl azide polymer arms. The terminal hydroxyl groups of a block copolymer of hyperbranched polyether and polyepichlorohydrin (**POE**) were tosylated, followed by azidation of the chloro and tosyl groups. With approximately 17,000 g mol^−1^, **POGA** had a significantly higher molecular weight than common plasticizers (200–1000 g mol^−1^), resulting in much slower exudation. Although its plasticizer efficiency was inferior to the commercially available small molecule plasticizer butyl nitrato ethyl nitramine (**bu-NENA**), it possessed a superior energy level and good safety performance [39].

## 3. Cross-Linking with Organic Azides (Part I): Organic Semiconductor Devices

### 3.1. Bisazides: The Forefathers of Photosensitizers in Lithography

The beginning of the 21st century has seen tremendous technical development of electronic equipment as a result of continued downscaling of semiconductor integrated circuits (**IC**s) This was only possible by the evolution of photoresist materials and lithography methods towards higher resolutions. Where the semiconductor industry had once started with ICs at the µm scale, it will soon be conquering the sub-7 nm region using extreme ultraviolet (EUV) lithography. At the beginning of this evolution, organic bisazides had the leading share as sensitizers in photoresist systems. In 1958, Eastman Kodak Co. filed a patent for an azide resin discovered by Kodak researchers Martin Hepher and Hans Wagner, also known as the “Kodak thin film resist” (KTFR) [2]. It was composed of the bisazide sensitizer 2,6-bis(4-azidobenzylidene)-4-methylcyclohexanone dispersed in a cyclized rubber (poly-*cis*-isoprene) and became the first working horse for semiconductor industry until it reached its resolution limit of 2 µm in 1972.

**Figure 2:**
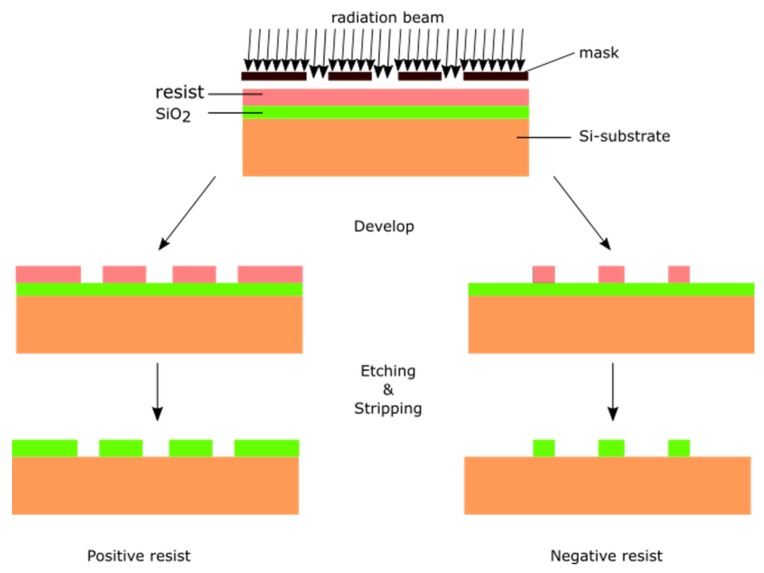
Principle of photolithography with positive and negative resists.

Commercial two-component photoresists for the fabrication of μm- and sub-μm structures in microelectronics technology are composed of a polymer resin and a photosensitizer. The principle of photolithography is depicted in Figure 2. A silicon wafer is first coated with an oxide layer. Then, either a positive or negative photoresist is deposited on it, dried, and exposed to UV light through a mask. Irradiation activates the crosslinking reaction between the photosensitizer and the polymer resin. This changes the solubility of the resin in the exposed region. In the final stripping process, depending on the type of photoresist, either the exposed (positive resist) or the unexposed (negative resist) area is soluble and is removed, leaving behind the desired pattern. Popular commercial negative photoresists consist of low molecular weight polymers derived from phenols and formaldehyde (so called novolacs) and aromatic bisazides as photosensitizers [40].

**Scheme 3:**
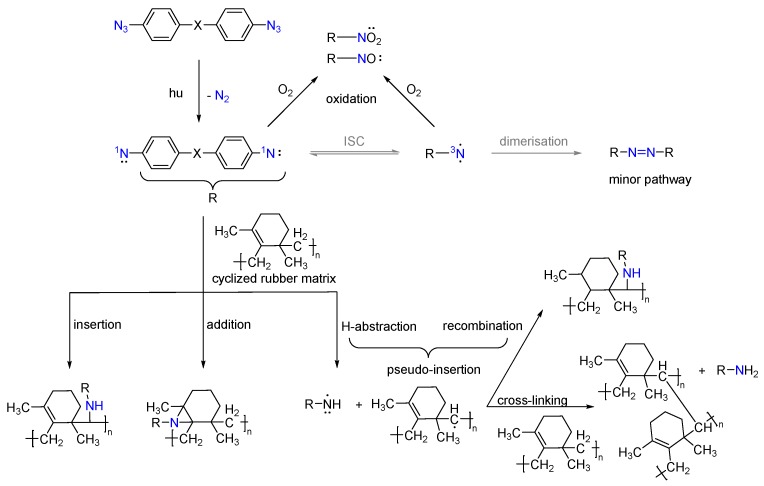
Crosslinking mechanism of bisazide photoresist systems. Reactions with triplet nitrene are depicted in grey.

Scheme 3 shows the chemical mechanisms in bisazide photoresist systems [40]. Irradiation of the photoresist with UV light generates highly reactive singlet nitrenes which can react with the polymer matrix creating covalent bonds via (1) insertion into C–H bonds, (2) addition to double bonds forming aziridine rings which is the most common way, and (3) radical abstraction followed by recombination of radical polymer chains. To a smaller extent, diradical triplet nitrenes are generated by fast intersystem crossing. It can form primary amines by double hydrogen abstraction and azo compounds by reaction with azide precursor or by dimerization at high nitrene concentration.

**Figure 3:**
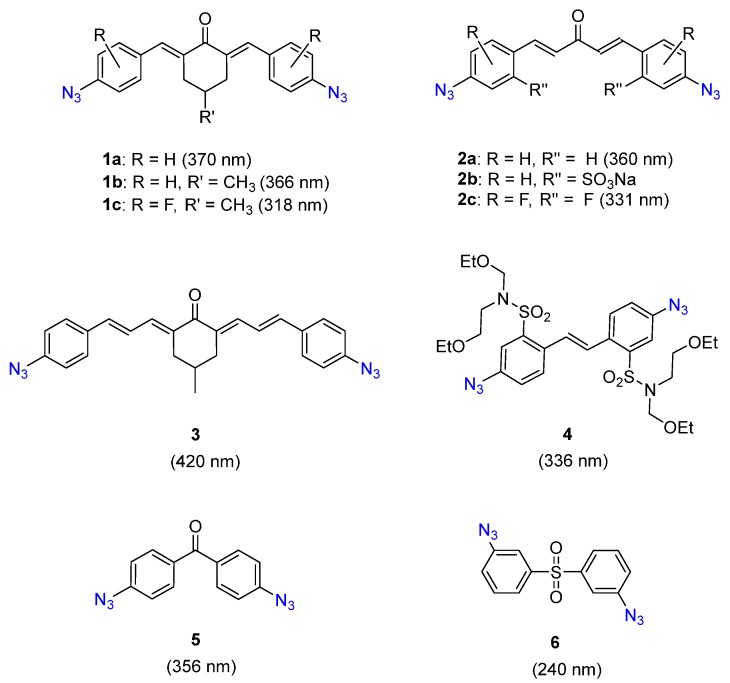
Bisazides used in photoresist systems. Absorption maxima are given in brackets (if available).

Both singlet and triplet nitrenes can be scavenged by oxygen (however this reaction is quite slow in comparison to insertion into C–H bonds). This is a disadvantage, because light exposure must be carried out under a nitrogen blanket or in a vacuum to prevent loss of photosensitivity. However, the rate nitrenes reacting with oxygen is slow compared to the reaction with carbon based radicals. Apart from a high quantum yield for photolysis (ϕ≥0.3), a good bisazide photosensitizer should have an absorption maximum adapted to the optimal working wavelength of the light source and good solubility in order to achieve high concentrations and thus the required film thickness for microstructuration. Various negative photoresist systems with different polymers like poly(methyl methacrylate) (**PMMA**) or poly(methyl isopropenyl ketone) (**PMIPK**) and bisazides like 3,3′-diazidodiphenyl sulfone or 2,6-bis(4′-azidolbenzylidene)-4-methyl cyclohexanone exist (see Figure 3) [41,42,43]. Most of these bisazides are activated by deep UV light between 250 nm to 300 nm, limiting their use as photosensitizers for polymer resins like poly(*p*-vinylphenol) (**PVP**) with strong absorption in this region [44,45]. Keana and co-workers found that perfluorinated bisazides can be crosslinked at the near UV region (around 350 nm) with high crosslinking efficiency for **PVP**, **PS**, **PMMA** poly(3-octylthiophene), and polyimide. For example, instead of 20 wt% of 3,3′-diazidodiphenyl sulfone, only 3.6 wt% of 1,5-bis(4′-azido-2′,3′,5′,6′-tetrafluorophenyl)-1,4-pentadiene-3-one (see Figure 3) were required to get 95% film retention after development of a **PVP** resist [46,47,48,49]. This was attributed to the enhancement of the C–H and/or N–H insertion reactions by the presence of fluorine atoms [50]. The lithographic performance of 2,2′-disubstitued diazidostilbene derivatives was optimized by Voigt on the basis of identified structure-property-functionality relationships [51]. At concentrations of 6–14 wt%, bisazide **4** (see Figure 3) had the best solubility in the series, allowing for a film thickness of up to 10 µm in combination with novolac resin. The bisazide had a moderate quantum yield of ϕ=0.26 (λ_exc_ = 313 nm) and an absorption maximum at 331 nm in methanol, rendering it suitable for lithography at a wavelength of 365 nm, called “i-line”, which is referring to the spectral line of the Hg lamps which had originally been used. The maximum resolution at this wavelength was 0.3 µm. A fully waterborne i-line lithography process for conducting thin films from poly(3,4-ethylenedioxythiophene) was developed by Touwslager et al. They used the water-soluble disodium salt of bisazide 4,4′-diazido-2,2′-disulfonic acid benzalacetone as a photoinitiator and were able to obtain all-polymer integrated circuits with a minimum feature size of 2.5 µm wide lines separated by 1-µm spacings [52]. Other examples in current research include fabrication of photo-patterned polymer-based field-effect transistors and light-emitting diodes and are presented in the following chapters.

### 3.2. Photovoltaics—Higher Performance with Locked Morphology

Compared to rigid, more fragile inorganic solar cells, the amenability to solution processed manufacturing methods of organic photovoltaics (OPV) such as ink-jet and roll-to-roll printing opens new possibilities for applications where flexibility is indispensable. Current key challenges are increasing performance and long-term stability. The main sources of device instability are thermal, chemical, or mechanical stress, and light exposure [53]. The bulk heterojunction (BHJ), an inter-percolating thin film structure of donor and acceptor materials between the electrodes, is the most common type of microstructure in OPVs. Current devices show a drastic decrease in their efficiency as a result of deleterious changes in the morphology of this microstructure. Crosslinking has been identified as a convenient method to control and stabilize BHJ morphology. The goal is to create a conjugated network of donor polymer with a frozen morphology in which the acceptor is embedded in order to prevent diffusion, aggregation and macrophase crystallization. This process should be activated without perturbing blend morphologies. Some cross-linkable groups require addition of initiators or produce reactive side products which can have negative impact on device performance and long-term stability. The azide functionality can be activated thermally or by UV light under formation of highly reactive nitrenes, which are capable of insertion and addition reactions (see Scheme 3). Popular BHJ blends consist of electron donating polymers like poly(3-hexylthio-phene-2,5-diyl) (**P3HT**) [54] or polybenzodithiophenes such as **PTB7 [55]** and fullerene derivatives like (6,6)-phenyl C_60_-butyric acid methyl ester (**PC60BM**) [54] as acceptor materials.

#### 3.2.1. Azido Polymers

Sidechains of conducting polymers like **P3HT** can be azido-functionalized for crosslinking of BHJ blends. For example, after UV crosslinking of a **P3HT-N5**/**PCBM** blend with 5% azide functionalized side-chains, inhibition of fullerene aggregation was observed. The device retained 65% of its power conversion efficiency (PCE) after comparatively mild ageing for 40 h at 110 °C compared to less than 30% retention using non-crosslinked commercial **P3HT** which underwent significant phase separation. However, the initial PCE of 1.5% was 0.7% lower than that of the reference, relativizing the higher stability achieved by crosslinking. This could be explained by limited charge separation arising from a compromised morphology or by degradation of the polymers under the harsh crosslinking conditions (60 min UV exposure in air) employed [56]. A comparative study with donor–acceptor-conjugated polymer **PBT** bearing photo-crosslinkable groups showed that an appropriate crosslinking network greatly improved the thermal stability of the BHJ cells. Devices fabricated with **PBT-Br/PCBM** and **PBT-N_3_/PCBM** blends that were photo-crosslinked by UV light exposure for 30 min retained 78% and 66% of their initial PCE of 2.1% and 1.8% after annealing for 80 h at 150 °C, respectively. Also, in this case, the initial PCEs were lowered by 0.6% and 0.9% for the bromo- and azido-polymer, respectively, after UV exposure, indicating some degradation of the blend materials [57].

#### 3.2.2. Small Molecule Azide Crosslinkers

**Figure 4:**
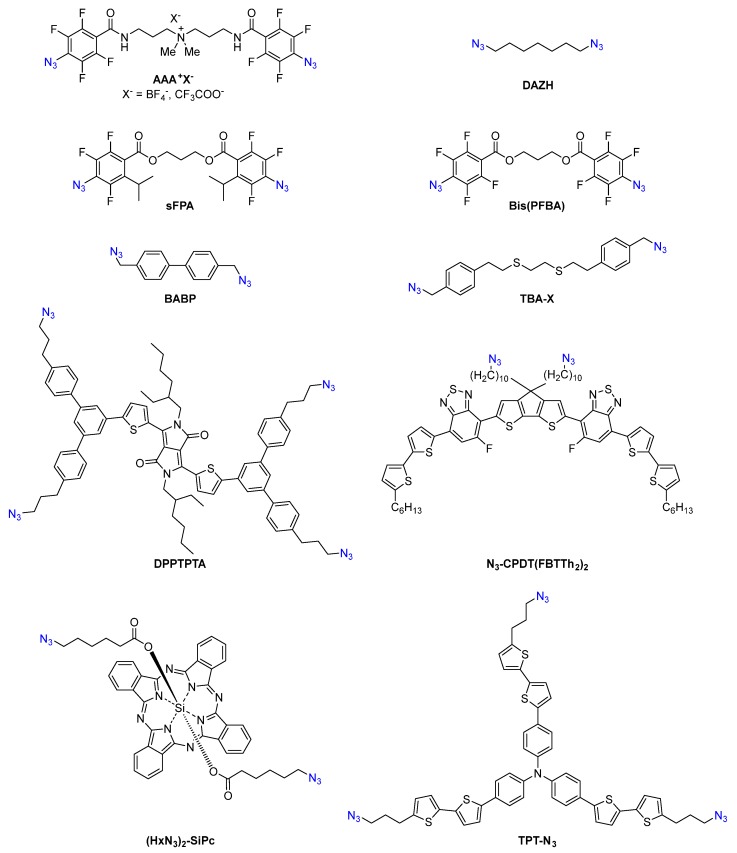
Bisazides used as crosslinkers in OPV: ionic 1,4-bis(perfluorophenyl azide) (**AAA^+^X**^−^), 1,6-diazidohexane (**DAZH**), ethylene bis(4-azido-2,3,5-trifluoro-6isopropylbenzoate) (**sFPA**), 4,4′-bis(azidomethyl)-1,1′-biphenyl (**BABP**), 1,2-bis((4-(azidomethyl)phenethyl)thio)ethane (**TBA-X**), ethylene bis(4-azido-2,3,5,6-tetrafluorobenzoate) **(Bis**(**PFBA**), 3,6-bis(5-(4,4″-bis(3-azidopropyl)-[1,1′:3′,1″-terphenyl)-5′-yl)thiophen-2-yl)-2,5-bis(2-ethylhexyl)-2,5-dihydropyrrolo(3,4-c)pyrrole-1,4-dione **(DPPTPTA)**, 4-4′-bis(1-azido)undecane)dicyclopenta-(2,1-b:3,4-b′)dithio-phene-bis(5-fluoro-7-(5′-hexyl-(2,2′-bithiophene)-5-yl)benzo-(c)(1,2,5)thiadiazole) N_3_-(**CPDT(FBTTh_2_**)_2_), bis(6-azidohexanoate)silicon phthalocyanine (**HxN_3_)_2_-SiPc**), and tris(4-(5′-(3-azidopropyl)-2,2′-bithiophen5-yl)phenyl)amine (**TPT-N_3_**).

Instead of using cross-linkable polymers, a fundamentally different approach is the use of small molecule crosslinkers as additives in BHJ blends. Several bisazides with both aliphatic and aromatic cores have been used in fullerene-type BHJ devices. As mentioned, crosslinking should proceed with high efficiency under mild conditions. The desired reaction of nitrenes is the insertion into alkyl C–H bonds, while attacks on the π-conjugated core of the polymer can degrade semiconductor properties. Png et al. found that sterically hindered bis(fluorophenyl azides) (**sFPAs)** show very good photo-crosslinking efficiency because C–H insertion is favored over side reactions. Only about 15% of the formed nitrenes insert into aromatic C–H bonds compared to 30% for unhindered bis(fluorophenyl azide) [58]. They crosslinked **P3HT** with bisazide **sFPA** (Figure 4) and then doped a **PC_61_BM** acceptor into the polymer network by solution spinning from chlorobenzene (**CB**). This method has potential for manufacture on scale as it can be implemented over large film areas and thicknesses. Because crosslinking renders the polymer film insoluble in the **CB**, a subsequent top acceptor layer could be solution deposited from the same solvent. The crosslinked device showed 20% improvement in PCE over conventional devices [8,59]. Similar results were obtained using bisazide 1,6-diazidohexane (**DAZH**) in combination with a polysilaindacenodithiophene-benzothiadiazole and (6,6)-phenyl-C_71_-butyric acid methyl ester **(SiIDT-BT/PC_71_BM)** blend. However, PCE losses upon thermal annealing or UV light exposure were significant in most cases, leaving room for improvement [60]. Another example of the superiority of azide crosslinkers in enhancing long-term stability of OPVs compared to other crosslinker types was presented by Chao et al. When crosslinking the active layer with an azide-functionalized triphenylamine derivative, **TPT**-N_3_, their device retained 75% of its initial PCE after extended thermal annealing for 144 h at 150 °C, whereas the bromo-derivative retained only 60%. The PCE of the reference device without crosslinker dropped to 0.24% under these conditions [61]. Jeng and co-workers synthesized the low-band gap bisazide cross-linkable bisazide **N_3_-(CPDT(FBTTh_2_)_2_)** with a D^1^-A-D^2^-A-D^1^-type architecture. This bisazide has a wide absorption range and can harvest additional solar light in addition to stabilizing the BHJ morphology. In contrast to other existing azide crosslinkers, full retention of the PCE after curing and aggressive thermal ageing at 150 °C for 24 h could be achieved at a ratio of 1:0.2:1 (**P3HT**:linker:**PC_61_BM**) [62]. However, residual unreacted hydroxy precursor contained in the target compound might have had an additional effect on this remarkable result.

**Figure 5:**
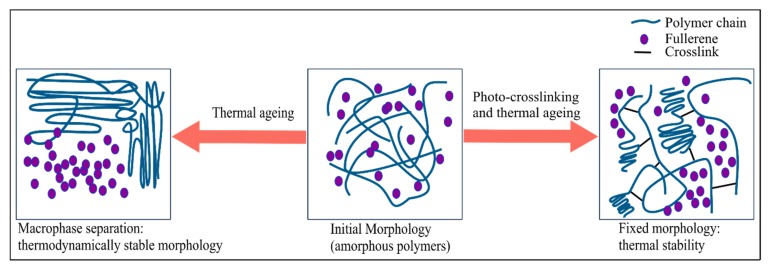
Left: Conventional thermal ageing of an amorphous bulk heterojunction (BHJ) blend (center) leads to macrophase separation over time. Right: Photo-crosslinking of the polymer prior to ageing fixates the chains in space and prevents fullerene aggregation.

In 2019, the same group reported for the first time on a tetravalent azide used as crosslinker. This multifunctional four-arm star-shaped diketopyrrolopyrrole-based conjugated small molecule, **DPPTPTA** has a broad absorption range, high charge mobility and can efficiently inhibit molecular aggregation. Incorporating 3 wt% of **DPPTPTA** into a **PTB7-Th**/**PC_61_BM** blend produced a significant change in the morphology of the blend film with well-defined D/A-separation, successfully suppressing crystallization of the **PCBM** acceptor. Ladder-type energy levels in the ternary blend might explain the improved charge separation and energy transport. The devices’ PCE was increased from 6.7 to 8.2% with 56% retention after thermal stressing at 150 °C for 18 h. Higher additive content was found to induce BHJ phase segregation, resulting in lower photovoltaic performance [63]. In the same year, Landerer et al. prepared a novel bisazide 1,2-bis((4-(azidomethyl)phenethyl)thio) ethane (**TBA-X**) in order to better control the crosslinking reaction and thus the BHJ morphology in fullerene-type BHJs with various donor polymers and fullerene acceptor **PC_61_BM** (Figure 5). Major advantages of **TBA-X** are its moderate reactivity and good solubility. Crosslinking could be initiated thermally already at a moderate temperature of 80 °C. Selective 1,3-dipolar cycloaddition with the fullerene took place at 150 °C affording triazolino (4′,5′:1,2) fullerenes. Nitrenes which could engage in random reactions with both the polymer donor and the fullerene were not formed at this temperature. The best-performing device based on a **PTB7/PC_61_BM** blend using 2 wt% crosslinker possessed a PCE of 6% with 90% retention after harsh thermal annealing of entire solar cells at 120 °C for 100 h [64]. An overview on the examples presented herein is given in Table 1.

In the realm of inorganic solar cells, so far only two examples of azide crosslinking exist. Watson et al. used the tetrahedral four-site crosslinking agent 1,3,5,7-tetrakis-(*p*-azidobenzyl)-adamantane (**TPBA**) to transform the soluble and fragile hole-transporting organic semiconductor poly(triaryl amine) (**PTAA**) into a solvent-resistant and mechanically tough film. Crosslinking also enabled fabrication of perovskite solar cells with increased photovoltaic efficiencies. The crosslinked polymer film showed better adhesion to the perovskite layer, mitigating interfacial device failure [68]. **TPBA** was also used by Watson et al. in the fabrication of a so-called compound solar cell, a perovskite solar cell partitioned into an array of microcells for increased chemical and mechanically stability [69].

### 3.3. Multilayered and Photopatternable OFETs and Organic Light-Emitting Diode (OLEDs)

**Figure 6:**
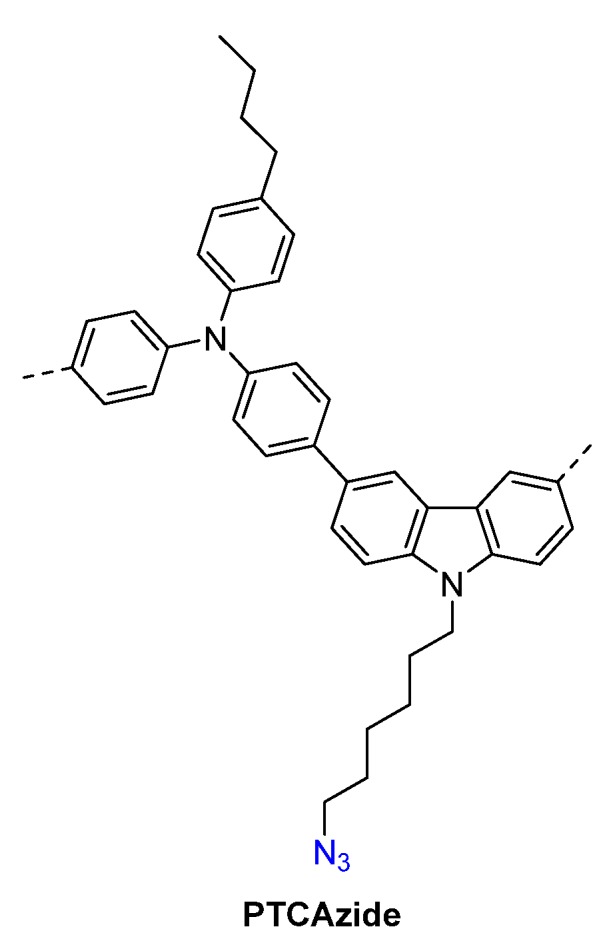
Chemical structure of (poly 4-(9-(6-azidohexyl)-9*H*-carbazol-3-yl)-N-(4-butylphenyl)-*N*-phenylaniline) (PTC).

Solution processes enable low-cost fabrication of large area multi-layer structured OLEDs. However, sequential deposition without dissolution of the underlying layer is challenging. One way to approach this issue is to transform solution-processed polymers into insoluble films by crosslinking. Park et al. designed a conducting copolymer from photo-crosslinkable poly(azido-styrene) (**PS-N_3_**) and hole-transporting material poly(triphenylamine) (**PTPA**). In contrast to harsh and lengthy thermal crosslinking, extremely short time (5 min) and low power UV light (2 mW/cm^2^, 254 nm) was enough to transform the polymers into insoluble films. Multi-layer OLEDs with a ratio of 5:95 of **PS-N_3_** to **PTPA** and a configuration of **ITO**/**PEDOT**:**PSS**/X-**PTPA**-5/**PVK**:**PBD**:Ir(ppy)_3_/LiF/Al performed twice as good as the control device without cross-linkable film. Furthermore, micro-patterned, pixelated OLEDs were fabricated through photolithography. The versatility of **PS-N_3_** to produce photo-crosslinkable, conducting films by copolymerization was exemplified using poly(carbazole) as one possible alternative hole-transporting material [70]. A different approach was made by Huang et al. Here, a 6-azidohexyl-functionalized carbazole was copolymerized with an alkylated triarylamine to obtain a cross-linkable hole-injection/transporting material named **X-PTCAzide** (Figure 6). Photo-crosslinking of a 15-nm-thick film was completed after 1 h with low power UV light (1 mW/cm^2^, 365 nm). A solution-processed electroluminescent device with the structure **ITO**/**X-PTCAzide**/Ir(ppy)_3_:**PVK**/**BCP**/Alq_3_/LiF/Al exhibited good performance with an external quantum efficiency of 7.93% and a luminous efficacy of 29.6 cd/A [71].

Modern applications of bisazides as photoresists were demonstrated in the fabrication of photopatternable OFETs and LEDs. For example, the bisazide ethylene glycol bis(4-azido-2,3,5,6-tetrafluorobenzoate) (**FPA**) allows crosslinking of polymers with high efficiency and is readily photo-patterned by deep UV and a shadow mask. It was used to produce a chemically robust dielectric film based on **PS** that provided a favorable environment for growth of a triethylsilylethynyl--anthradithiophene (**TES-ADT**) semiconductor film, yielding OFETs with much higher field-effect mobility than those with plain **PS** layers [72]. The sterically hindered bis(fluorophenyl azide) **sFPA** (Figure 4), was used to fabricate a photo-patterned poly(9,9-dioctylfluorene-alt-benzothiadiazole) (**F8BT**) film. In addition, a stack of eight alternating 50-nm-thick films of poly(9,9 di-n-octylfluorene-alt-(1,4-phenylene-((4-sec-butylphenyl)imino)-1,4-phenylene) (**TFB**) and **F8BT** was realized by sequential deposition and photo-crosslinking of the individual polymer layers. In the fabrication of LEDs with the structure **ITO/PEDT:PSSH/TFB/F8BT/Ca/Al**, this technique allowed fine control of the **TFB/F8BT** heterostructure simply by spin-casting **TFB** on poly(3,4-ethylenedioxythiophene) doped with poly(styrenesulfonic acid) (**PEDT:PSSH**) instead of the previously needed hard-bake procedure. With a 10-nm-thick **TFB** interlayer, the efficiency of the **F8BT** light-emitting film jumped tenfold to 4.4%. Moreover, both hole and electron **OC_1_C_10_-PPV** diodes with various crosslink densities showing little hole or electron trapping were fabricated. Because the electronic transition of the **sFPA** does not interfere with transitions of many polymer OSCs, deep UV exposure into polymer films of up to 300 nm thickness is possible with low risk of polymer photo-oxidation [58]. A common problem in OSC devices is electromigration of the conducting polymer. For example, devices fabricated by Png et al. using **PEDT:PSSH** as hole-injection layer (HIL) showed electromigration of **PEDT^+^** cations towards the OSC interface. By crosslinking of the HIL with water-soluble ionic 1,4-bis(perfluorophenyl azide) **AAA^+^X^−^** (Figure 4), migration was successfully suppressed in unipolar hole-only diodes. Although **PEDT^+^** electromigration was found to be a minor contributor to the voltage rise in these diodes, it may be important in bipolar OLEDs. Here, a high **PEDT** concentration at the HIL/OSC interface could promote exciton quenching and prevent recombination of the electrons in the light-emitting OSC by facilitating electron escape [73].

### 3.4. Stretchable Polymer Semiconductors

**Figure 7:**
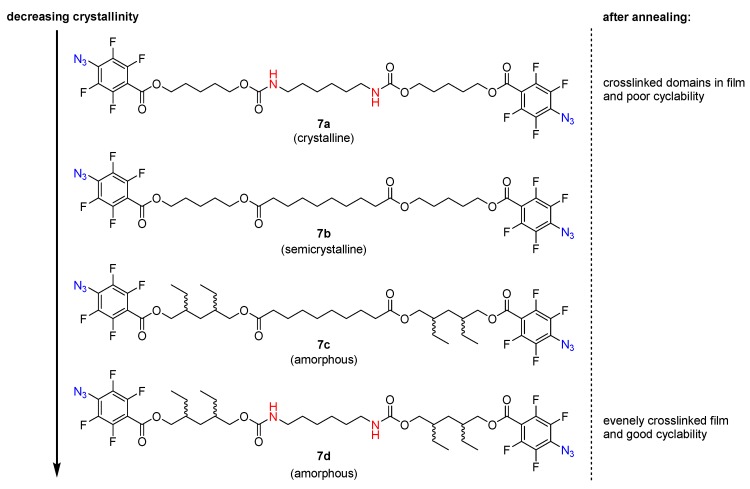
Influence of crosslinker morphology on film morphology.

The growing popularity of wearable and implantable electronics has stimulated the evolution of intrinsically stretchable polymer SCs. These can be obtained by crosslinking of elastomers. Recently, the importance of the crosslinker crystallinity on the mechanical properties of the polymer SC was highlighted. Wang et al. were able to control the crystallinity of alkyl bridged perfluorophenyl bisazides **7a**–**d** by introducing different functional groups into their alkyl bridge (Figure 7). Incorporation of urethane groups encouraged self-assembly via H-bonding, thus yielding a crystalline crosslinker. Ester groups gave semi-crystalline material due to the absence of H-bonding. On the other hand, creating tertiary carbon centers with ethyl side chains suppressed crystallization by steric hindrance. All bisazides had good thermal stability and activation temperatures for nitrene formation at around 172 °C. Only with branched, amorphous crosslinker **7d** evenly crosslinked elastic films with good cyclability could be obtained (Figure 5). Fully stretchable bottom-gate-top-contact OFET devices with crosslinked **DPP-10C_5_DE** layers were fabricated. While the initial field-effect hole mobility μ_h_ of ∼0.25 cm^2^V^−1^s^−1^ of the control device with neat polymer decreased with an increase in strain, the crosslinked films displayed stable μ_h_ values in the parallel to strain direction. Charge transport in the direction perpendicular to strain was significantly lowered because the polymer chains aligned in the strain direction. Those devices with polymer films crosslinked with crystalline crosslinker showed a significant drop in μ_h_ in the perpendicular direction after being subjected to 5000 cycles. This was attributed to the film’s high surface roughness leading possibly to delamination between the layers. Contrastingly, films crosslinked with the amorphous bisazide exhibited stable hole mobilities in most cases [74].

## 4. Cross-Linking with Organic Azides (Part II): Exchange Membranes

### 4.1. Azides in Proton Exchange Membranes

**Figure 8:**
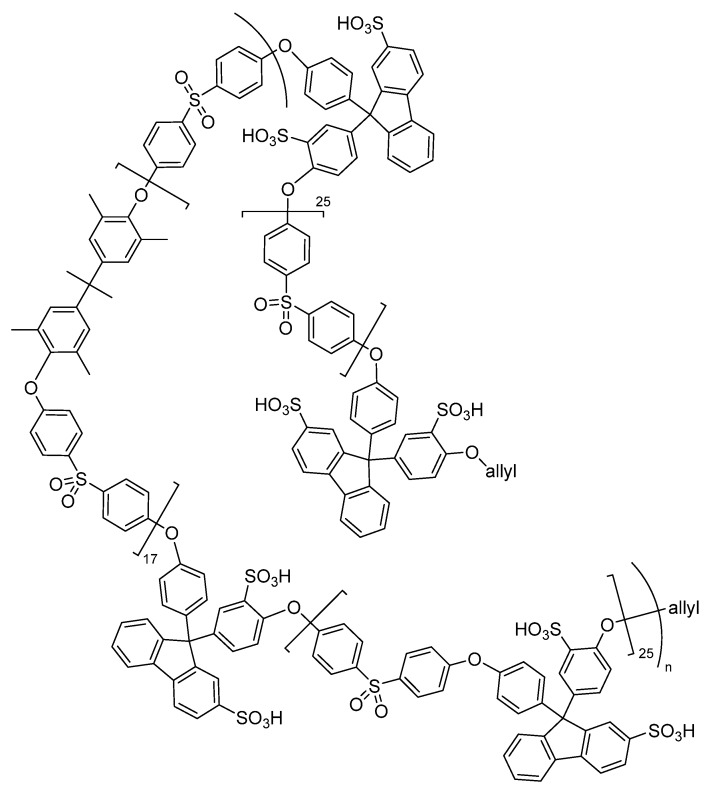
Structure of the sulfonated poly(ether sulfone) (SPES)-based ionomeric block copolymer with terminal allyl groups for crosslinking.

Apart from solar cells, proton exchange membrane fuel cells (**PEMFC**s) and direct methanol fuel cells (**DMFC**s) are very promising future power sources. Nafion^®^, a perfluorosulfonated polymer, is currently the most commonly used polymer electrolyte membrane in these devices, but due to its high cost and poor performance at high temperature, new polymers are sought. High proton conductivity *σ* is achieved with low methanol permeability and hydrophilic character of the polymer. However, hydrophobic membranes have higher physical stability. Finding the right balance has proven to be difficult. Polymer crosslinking with organic azides can effectively control the hydrophobic nature of the membrane as shown in the following examples. Kim and co-workers have used 2,6-bis(4-azidobenzylidene)-4-methyl-cyclohexanone, a popular crosslinking agent in photolithography (see chapter 3.1), to crosslink sulfonated poly(ether sulfone) (**SPES**) at allyl *termini* by thermal activation. Compared to Nafion^®^-112 (*σ* = 0.48 S/cm), the crosslinked membrane had an excellent proton conductivity of 0.79 S/cm at 100 °C and a much lower methanol permeability of 5.2 × 10^−7^ cm^2^s^−1^, corresponding to only 17% of that of Nafion^®^. The membrane withstood oxidation by Fenton’s reagent for up to 4 h compared to around 3 h for the non-crosslinked version [75,76]. The same bisazide was also tested in combination with a poly(fluorenyl ether sulfone) polymer, resulting in lower proton conductivity of 0.38 S/cm at 100 °C, but also even lower methanol permeability of 3.9 × 10^−8^ cm^2^/s. In both examples however, the proton conductivity at 20 °C was still lower than that of Nafion^®^. Therefore, the group applied their terminal crosslinking approach to a **SPES**-based ionomeric block copolymer with defined blocks. Sulfofluorenyl moieties were incorporated to increase water uptake and proton conductivity (Figure 8). This membrane outperformed Nafion^®^ at temperatures above 40 °C, reaching a value of 0.38 S/cm at 100 °C [77].

**Scheme 4:**
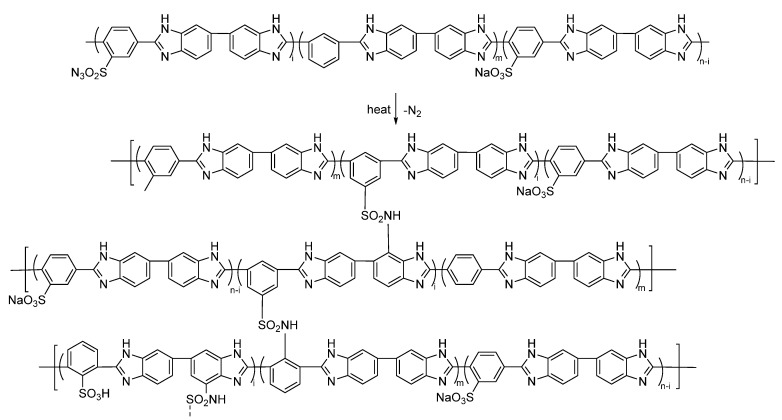
Crosslinking reaction of polybenzimidazoles with sulfonyl azide groups for proton conducting membranes.

A common feature of these terminally crosslinked membranes is their high temperature dependence compared to the non-crosslinked versions or Nafion^®^. The authors believe “that the hydrothermal energy on heating caused a positive structural change, leading to a phase separation for the crosslinked membrane, and this facilitated the crosslinked membrane to overcome a high activation energy (26.49 kJ mol^−1^) for proton conduction.” [78] A crosslinked membrane suitable for high-temperature PEM fuel cells was presented by Papadimitriou et al. It is based on aromatic polyethers with cross-linkable propenyl groups as side chains. For crosslinking, 25–30 wt% 2,6-bis(4-azidobenzylidene)-4-methyl-cyclohexanone (**1b**) (Figure 3) was thermally activated to react with the propenyl side-chains forming aziridines and amines. After a major weigh loss between 330 °C and 500 °C due to decomposition of nitrene intermediates, the crosslinked membrane showed superior thermal stability up to 760 °C compared to the non-crosslinked version. Crosslinking is known to lead to more compact structures, thus decreasing the material’s doping ability and its conductivity. Interestingly, the crosslinked membrane showed slightly higher doping levels (160 wt% H_3_PO_4_) and conductivity (0.088 S/cm) than the neat polymer (195 wt% H_3_PO_4_, σ = 0.062 S/cm), which could be attributed to favorable interactions between the phosphoric acid (**PA**) and the aziridine and amine functionalities formed during crosslinking. During preliminary fuel cell tests the membrane delivered stable performance at 200 °C for 48 h [79]. Instead of crosslinking with bisazides, Su et al. introduced sulfonyl azide groups into polybenzimidazole (**PBI**). Upon heating the PBI chains were cross-linked via the insertion reaction of generated sulfonyl nitrenes into the Ar–H bonds of the benzimidazole units. These crosslinked membranes (see Scheme 4 for the structure) had improved migration stability of **PA**, chemical oxidative stability and improved tensile strength, whereas the doping ability with **PA** and the proton conductivity decreased, reaching around 10^−4^ S/cm at 190 °C with 20% **PA** content [80].

### 4.2. Anion Exchange Membranes

Until now, very few examples of crosslinking with azides for fabrication of anion exchange membranes (AEMs) exist. Tough and flexible AEMs based on poly(2,6-dimethyl-phenylene oxides) (**PPO**) with azido-functionalized side chains were produced by thermally activated crosslinking via nitrenes. Illustrating the versatile reactivity of the azide function, the side chains were also used for quaternization of the polymer by CuAAC with an alkyne-functionalized ammonium precursor. The authors found that, despite having a decreased water uptake, their AEMs based on nitrene crosslinking had substantially higher hydroxide conductivities of up to 57.7 mS/cm compared to AEMs known in literature using other crosslinking techniques. This could be attributed to the architecture of the sidechains, providing a continuous conduction pathway between the 1,2,3-triazoles bearing a quaternized ammonium function and water molecules/hydroxide ions [81]. He et al. synthesized a quaternized polysulfone with azide side groups for thermal crosslinking with reduced graphene (**rGO**) as inorganic nanofiller. At 160 °C, the azide groups reacted with alkenyl groups of the **rGO** under formation of aziridines. In comparison with the pristine membrane, the crosslinked membranes showed better mechanical strength and thermal stability. Moreover, improved alkaline resistance was observed. This might be the result of the crosslinked structure impeding access of the hydroxide ions to the interior of the membrane. On the downside, crosslinking adulterated water uptake and led to the formation of smaller ionic clusters in the membrane, thereby impeding continuous ion transport. However, using as low as 0.1 wt% **rGO** already greatly lowered the methanol permeability to 1.38 × 10^−9^ cm^2^/s and enhanced the ion selectivity by the order of two magnitudes from 7.56 × 10^5^ to 1.21 × 10^7^ S/cm^3^, surpassing commercial Nafion^®^-117 by far [82].

## 5. Cross-Linking with Organic Azides (Part III): More Applications

### 5.1. Long-Chain Branching in Polyolefins

Long-chain branching (LCB) in polyolefins can enhance melt strength and improve processing properties. However, metallocene polyethylene (**PE**) [83], the most widely used polymer nowadays, is linear and has a narrow molar mass distribution. The resulting narrow processing window is disadvantageous for industrial application. LCB of **PE** was achieved by thermal crosslinking with 1,3-benzenedisulfonyl azide (**1,3-BDSA**) [6]. At concentrations above 100 ppm of **1,3-BDSA**, LCP effects became evident: the zero shear viscosity increased from 2.8 kPa s with 128 ppm **1,3-BDSA** to 45 kPa s with 1027 ppm **1,3-BDSA**. The steady state compliance J_e_^0^ increased from 0 to 512 ppm, but then decreased at higher concentration, which might be attributed to a change of the dominant shape from linear to star shape. The bisazide crosslinker had a good efficiency between 40–60% and low propensity for chain scission reaction. Therefore, this LCB approach is a viable alternative to copolymerization of **PE** with 1-olefins.

### 5.2. Dynamic Vulcanization

**Figure 9:**
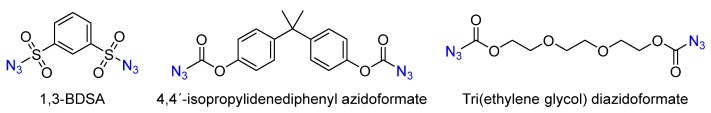
Bisazide crosslinkers for dynamic vulcanization.

**1,3-BDSA** has also been used as crosslinking agent for dynamic vulcanization of ethylene–propylene–diene terpolymer rubber (**EPDM**) and polypropylene blends to produce thermoplastic vulcanizates (TPVs). The TPVs belong to the larger family of thermoplastic elastomers (TPEs). They can be processed by conventional thermoplastic fabrication processes but exhibit more elastomer-like properties than mechanical blends, making them interesting materials. This is a result of the high degree of crosslinking of the elastomeric chains. Vulcanization of the elastomer takes place during its intimate melt mixing with the thermoplastic and leads to a fine dispersion of crosslinked small rubber particles in the thermoplastic matrix. In contrast to peroxides, curing with **1,3-BDSA** does not give rise to chain scission of the matrix. **1,3-BDSA** was found to be a better vulcanization agent than elementary sulfur, as evidenced by improved rheological and mechanical properties. Because of its ability to undergo C–H insertion reactions, it does not only efficiently cross-link the elastomer phase but also creates a higher number of covalent links to the thermoplastic phase, thereby affording more rigid and stable three-dimensional networks [3]. This mechanism was also proposed in another study, where **1,3-BDSA** was compared with two bisazidoformates (see Figure 9) for **PP/EPDM**-based TPVs from the perspective of processability and material properties. In contrast to all other curing systems tested, bisazides lead to a significant reduction of the rubber particle size with increasing crosslink density. Low **1,3-BDSA** content gave the best processability of the vulcanizate. Moreover, **1,3-BDSA** was more suitable for curing than the azidoformate compounds, which were too reactive during **iPP/EPDM** melt mixing due to their lower decomposition temperature.

In another example, 1,12-diazido-dodecane was employed in the crosslinking of poly(dimethylsiloxane) (**PDMS**) elastomers and coatings. This material exhibits high temperature and chemical resistance as well as properties making it interesting in food and biomedical industries. Most frequently used curing systems are, however, based on metal catalysts, thus preventing their application in these field due to possible metal contamination. Free radical curing with organic azides circumvents this issue. At 90 °C, the azide reacts with the vinyl-side chains of **PDMS** to form two main coupling products: 1,2,3-triazolines by 3 + 2 cycloaddition and aziridines by rearrangement of 1,2,3-triazolines or by radical addition of nitrenes issued from thermal decomposition. By this method, highly crosslinked films could be obtained, although only 10–20% of bisazide molecules formed elastically active strands. The crosslinking density was lower at higher temperatures, probably due too side reactions resulting from an excess of reactive nitrene radicals as well as evaporation of the bisazide [84].

### 5.3. Nanoparticles

**Scheme 5:**
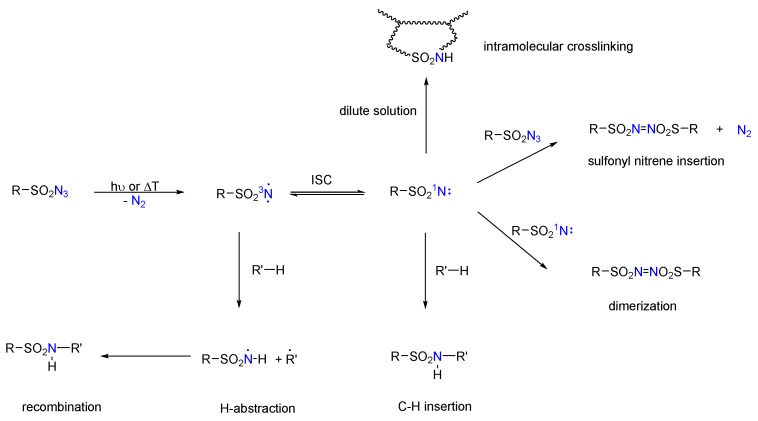
The crosslinking mechanisms of sulfonyl azides are akin to those of aromatic or aliphatic nitrenes (Scheme 3).

The versatility of bisazides in crosslinking reactions was highlighted by the preparation of well-defined polyester nanoparticles via intermolecular chain collapse using the CuAAC reaction. By varying the number of alkyne sidechains in the linear polyester precursor and the amount of bisazide crosslinker 1,8-bisazide-3,6-dioxaoctan, the size of the nanoparticles could be controlled. For example, using a polyester with two alkyne sidechains per 17 repeating units and four equivalents of azide per alkyne moiety yielded a particle size with 178 nm diameter. Doubling the amount of azide roughly doubled the particle diameter. Disadvantages of this CuAAC approach were the long reaction time of 24 h and possible Cu(I) contamination of the product [85]. To avoid this, Jiang et al. used sulfonyl azide functionalized linear copolymers that form nitrenes upon heating, which insert into the C–H bonds of the backbone and lead to intramolecular crosslinking in dilute solution. The binding mechanisms of sulfonyl azides (Scheme 5) are akin to those of aromatic or aliphatic nitrenes (Scheme 3). Advantages over thermal crosslinking using benzocyclobutene (**BCB**) are the lower reaction temperature of 180–200 °C vs. 250 °C and the higher reactivity of the nitrene species. The nanoparticles’ diameter could be controlled by the content of sulfonyl azide groups and by the molecule weight of the polymer [86].

**Scheme 6:**
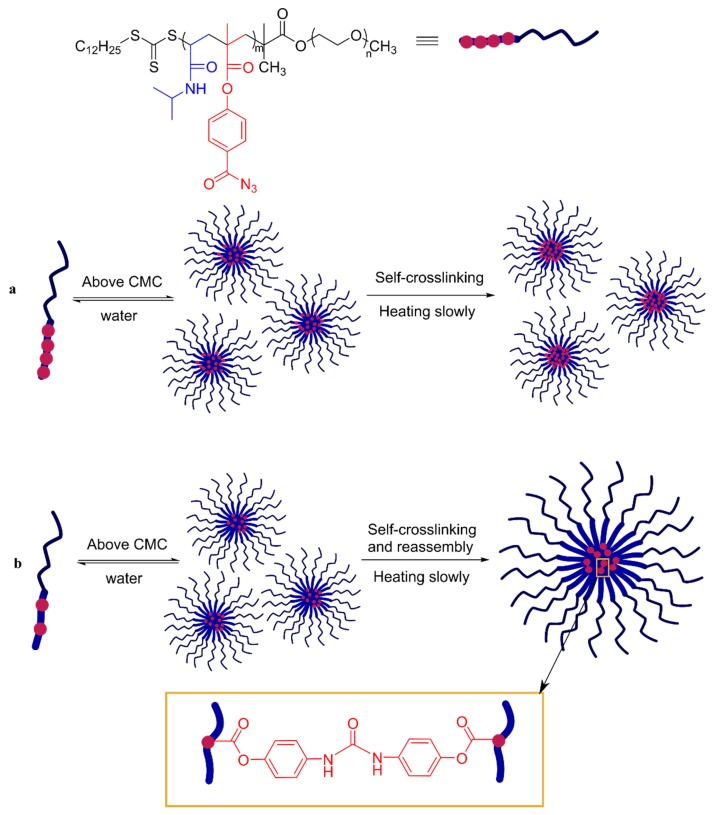
(**a**) Assembly of block copolymer **PEG-*b*-P(NIPAM-*co*-AAPMA)** chains at critical micelle concentration (CMC) followed by heat induced self-cross-linking into sub-20 nm micelles takes place above a certain concentration of acyl azide groups in the polymer. (**b**) Otherwise, reassembly into larger units occurs. Scheme adapted from [89].

A promising class of nanoparticles (NPs) for drug delivery and imaging are block-copolymer nanoparticles because they can be functionalized both at the core and the surface. To increase the throughput of massively parallel signature sequencing (MPSS), an imaging method for DNA sequencing, Zhang et al. synthesized core-crosslinked NPs as a replacement for larger, micro-scale latex beads. They crosslinked the core of polystyrene-block-poly(ethylene oxide) NPs via nitrene insertion by photolysis of encapsulated aryl bisazides 1,4-diazidobenzene (**DAB**) and 4,4′-bis(azidomethyl)-1,1′-biphenyl (**BABP**). When using **BAPB**, NPs exhibited improved thermal stability, while **DAB** did not function well in this regard. The authors attribute this to the different intramolecular distance of the azide moieties: the longer BABP might more readily span the distance between two PS chains within the core of a NP. Nanoparticles that were core-crosslinked with **BABP** did not shrink in size under mock polymerase chain reaction (PCR) conditions and could therefore serve as nanobeads for PCR [87] Instead of using small molecule crosslinkers, Hu et al. prepared NPs by self-assembly of an amphiphilic block copolymer containing acyl azide groups in a one-pot reaction in aqueous media. The block copolymer **PEG-b-P(NIPAMco-AAPMA)** was synthesized by RAFT copolymerization of *N*-isopropylacrylamide (**NIPAM**) and 4-(acyl azido) phenylmethacrylate (**AAPMA**) in the presence of macromolecular chain transfer agent PEG5000-CTA at room temperature. In contrast to nitrene and click chemistry of alkyl or aryl azides, acyl azides show a different crosslinking mechanism. Upon heating, they transform into isocyanates via Curtius rearrangement, which readily react with water to form amino groups [88]. Subsequently, crosslinking between the amino groups and unreacted isocyanate groups can occur. Above a certain concentration of acyl azide groups in the copolymer, self-crosslinking without reassembly into bigger micelles was observed and NPs with a diameter of as low as 17 nm were obtained (Scheme 6) [89]. While the preparation of sub-20 nm particles often is cumbersome, the strategy presented here is convenient, economical and environmentally benign. Moreover, it can be useful for the preparation of hollow polymer nanospheres, an emerging class of material with exciting properties [90].

### 5.4. Hydrogels

**Scheme 7:**
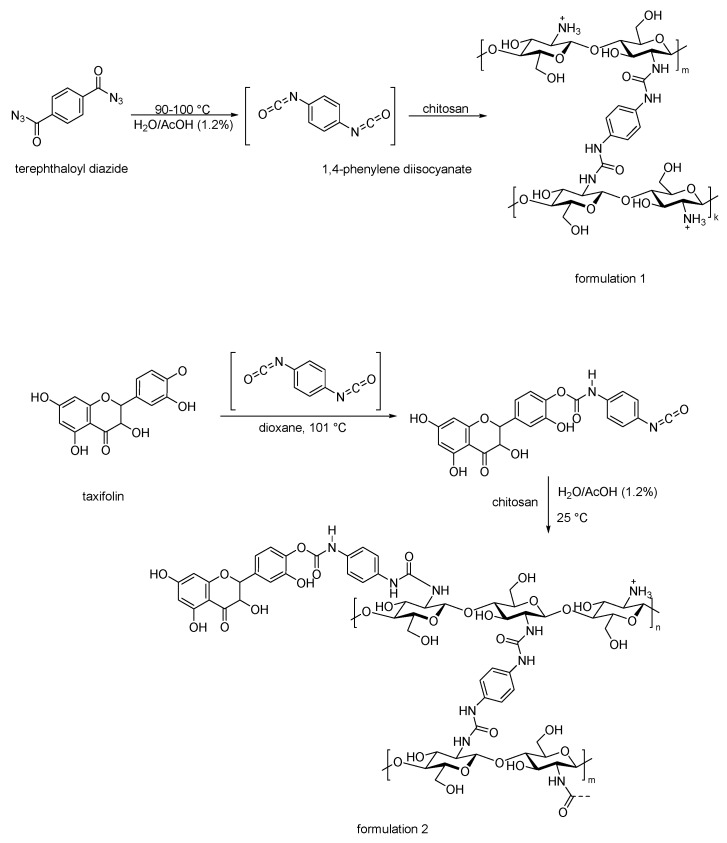
Chitosan-based drug release systems using terephthaloyl bisazide as a crosslinker.

Hydrogels are highly water-absorbent three-dimensional networks of crosslinked polymer chains. They can be designed as responsive materials which upon stimulation by external factors can liberate specific compounds. A pH- and thermosensitive hydrogel based on chitosan crosslinked with terephthaloyl bisazide was synthesized by Odinokov et al. as a drug delivery system. Crosslinking was achieved by nucleophilic addition of polysaccharide amino groups to 1,4-phenylene diisocyanate generated in situ by thermolysis of the bisazide (see Scheme 7). Inclusion of the bioactive compound taxifolin was provided by formation of hydrogen bonds with the chitosan network. In a second formulation, taxifolin was covalently linked to the chitosan network via the second isocyanate group of the adduct formed between taxifolin and 1,4-phenylene diisocyanate. Increasing the content of crosslinker for hydrogel formation led to a decrease of taxifolin release.

With 0.05 mmol bisazide per 20 g of a 3% solution of chitosan, more than 80% of the drug was released from the hydrogel within 12 h, while only 49% was released with glutaric aldehyde as a crosslinker. In case of the second formulation, enzymatic hydrolysis released less than 3% of taxifolin within 28 h [91].

### 5.5. Porous and Gel-Like Materials with Azides

**Figure 10:**
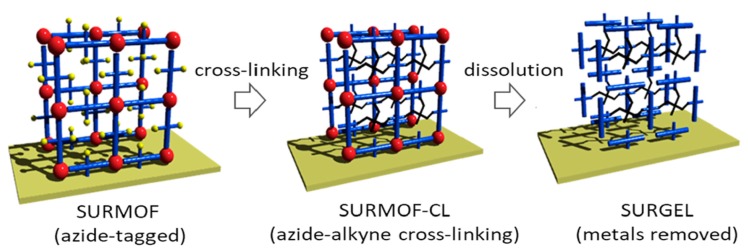
Transformation of azide-tagged SURMOF into surface-anchored polymeric gel (SURGEL) via azide-alkyne cross-linking and subsequent metal removal after dissolution [96].

Due to their high specific surface areas, well-defined pore sizes, and functional sites, porous materials are employed in molecular adsorption, storage and separation, sensing, catalysis, energy storage, and conversion and drug delivery [92]. Rigid polyazides with tetrahedral cores have been used for the synthesis of porous materials: For example, the fourfold click reaction on tetraphenylazido methane or tetraphenylazido adamantane with 1,4-diethynylbenzene yielded hyper-cross-linked polymers (HCPs) in good yield. Adsorption measurements of N_2_ and CO_2_ suggest extensive microporosity with a pore size of around 2 nm as well as presence of pores as large as 8–10 nm which are “likely due to interparticle cavities generated by the aggregation of nanoparticles.” [93] In addition, fullerenes have been used for similar purposes [94,95]. Moreover, surface-anchored MOFs can be functionalized with azide groups and subjected to cross-linking with alkynes. Dissolution yields surface-anchored polymeric gels (SURGELs) (Figure 10). These water-stable and metal-free nanoporous polymer materials may be functionalized postsynthetically photo-patterned and precisely structured at different length scales from sub-nm to cm appropriate to bioactive functions. High biocompatibility and stability render them suitable for biotechnological applications [96].

## 6. Polymerization Reactions with Azides

### 6.1. The aza-Wittig Polymerization towards Poly(azomethine)s

Poly(azomethine)s are a special class of π-conjugated polymers because they contain imine double bonds with a free electron pair on the nitrogen atom that can interact with many electrophilic substances and chelate metal ions. The most salient properties of these poly(Schiff-bases) are their excellent thermal stability, electrical conductivity, and second- and third-order nonlinear optical properties. As it is impossible to control the reactivity of the conventional polycondensation between amines and aldehydes, Chujo and Miyake reported another polymerization method towards poly(azomethine)s utilizing the aza-Wittig reaction. In this nitrogen-containing version of the Wittig reaction, *N-P*-ylides, formed from organic azides and phosphines, react with carbonyl compounds to give imines under mild conditions [97,98]. The reactivity of the aza-Wittig polymerization could be easily tuned by changing the steric and electronic features of phosphines employed. In the presence of tri-*n*-butylphosphine, 1,4-diazidobenzene and 2,5-bis(3,7-dimethyloctyloxy)terephthalaldehyde readily underwent an A_2_B_2_-type polycondensation reaction to yield a soluble π-conjugated all-*trans* poly(azomethine) with high molecular weight of *M*_n_ = 31,000 Da and a polydispersity of PDI = 2.03 [99]. The authors have also synthesized fully regioregular, head-to-tail coupled poly(azomethine)s from AB-type monomers containing both the aldehyde and azide functionality. Remarkably, self-condensation was not an issue in the aza-Wittig reaction. X-ray diffraction measurements indicated a highly crystalline layered order structure of the regioregular polymers, while the absence of any diffraction peak indicated the amorphous structure of the regiorandom version. The high crystallinity could lead to better π–π-stacking and elongation of conjugated length, which is indicated by a greater absorption at higher wavelength. The solution processability and crystallinity of these polymers makes them interesting for optoelectronic device applications including organic field effect transistors and photovoltaic cells [100]. However, their low solubility in toluene resulted in early termination of the polymerization and low molecular weights. Therefore, poly(azomethine)s were end-functionalized with polyhedral oligomeric silsesquioxanes (**POSS**) units to increase solubility. This did not only result in an increase of the molecular weight from 12,000 g mol^−1^ to 19,800 g mol^−1^, but also in enhanced electroluminescence and thermal stability of the devices prepared with this polymer. Moreover, the kinetic advantage of the aza-Wittig polymerization over the conventional polycondensation reaction was demonstrated by almost complete consumption of the end-capping unit within 2 h, while the conventional polycondensation reaction remained at about 40% conversion even after 4 h reaction time [101].

### 6.2. The Staudinger Reaction towards Polyphosphazenes

Polyphosphazenes are organic–inorganic hybrid polymers with P═N repeating units. They exhibit high thermal and chemical stability, flame resistance and remarkable elasticity and are used in drug delivery, biosensors, superhydrophobic surfaces and tissue engineering. The most popular synthesis route via ring-opening polymerization requires high temperatures and vacuum which may degrade the polymer [102]. In contrast, the Staudinger reduction offers a mild route to polyphosphazenes using phosphines as azide reducing agents. In the first step, a reactive phosphazide is formed by nucleophilic attack of the phosphine to the azide. The phosphazenes resulting from subsequent loss of nitrogen are usually prone to hydrolysis yielding amines, which are desired target products of the Staudinger reduction [98]. However, depending on the starting materials, stable polyphosphazenes can be obtained. Sundhoro et al. found that the reaction between perfluoroaryl bisazides and certain bisphosphines with aromatic or aliphatic linkers yielded polyphosphazenes with molecular weights close to 60,000 g mol^−1^ and dispersities as narrow as 1.1. This was a significant improvement over polyphosphazenes prepared with non-fluorinated bisazides. Moreover, the novel polymers showed high thermal stability with decomposition temperatures up to 441 °C and char yields up to 53% [103].

### 6.3. Multicomponent Reactions with Azides

**Scheme 8:**
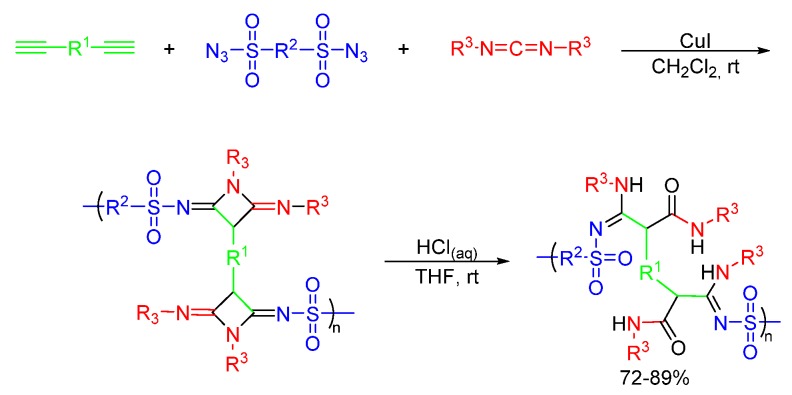
Synthesis of multisubstituted poly(azetidine)s via copper-catalyzed multicomponent polymerization reaction and subsequent acid-mediated ring-opening reaction.

Multicomponent reactions (MCRs) are one-pot reactions with three or more reactants leading to highly selective products while retaining the majority of the atoms of the starting material. MCRs show good functional-group tolerance, high atom economy, and simple isolation procedures. Similarly, functional polymers can be generated in multicomponent polymerizations (MCPs) with the appropriate choice of monomers. Based on the copper-catalyzed MCR of terminal alkynes, sulfonyl azides, and carbodiimides by Xu et al. [104], Han et al. designed an MCP to produce functional polymers featuring multisubstituted azetidine rings in the polymer backbone. The reaction was catalyzed by copper(I) iodide and conducted in dichloromethane at room temperature, obtaining polymers with high molecular weight of up to 29,200 g mol^−1^ in good yields (72–89%) (see Scheme 8). Polymers containing tetraphenylethenyl (**TPE**) moieties showed aggregation-induced emission with fluorescence quantum yield of up to 27.1% in solid state. Surprisingly, the solid powders of then two polymers without **TPE** fluorophores exhibited visible light emission under 362 nm UV. The crystal structure of a model compound suggests strong intramolecular through-space π-π and other electronic interactions which was verified by DFT calculations. New polymers with distinctive properties were produced by transforming the four-membered azetidine rings into amide and amidine moieties via acid-mediated ring-opening reactions in a mixture of aqueous HCl solution and THF. All polymers possess good film-forming ability and good photo-patternability, making them promising materials in electronic and optoelectronic devices [105].

## 7. Summary

The first chapter on high-energy materials makes it clear that organic polyazides should be handled with care, but they show also ample opportunities. As nitrogen-rich class of compounds, they are employed in explosive mixtures and are prone to violent decomposition upon stimuli such as light, heat, impact, and friction. However, the examples presented herein show that many compounds can be handled safely. **GAP** is a popular commercial energetic binder and the family of azido esters has been shown to be a green alternative to nitrate ester plasticizers, with better compatibility with azido binders, from the first application of bisazides in photoresists in semiconductor production to morphology control in organic photovoltaics. In material sciences, organic polyazides have been established as highly efficient cross-linking agents. Two classes of organic polyazide cross-linkers can be distinguished: small molecule crosslinkers and azido polymers. A major advantage of organic polyazide cross-linkers is that they can be both thermally and photo-activated without additive. Cross-linking proceeds either via heat- or light-induced decomposition of the azides into highly active nitrenes, which readily react with both saturated and unsaturated hydrocarbon chains or via cycloaddition reactions with alkynes. The orthogonality of these approaches opens up even more possibilities in cross-linking materials. This has led to the development of interesting new materials in the field of life sciences, such as cross-linked hydrogels for drug delivery. Finally, exploiting the diverse reactivity of the azido group employing organic polyazides with novel polymers provided a number of new features. Using multicomponent reactions as well as more convenient alternative routes led to polydiverse novel polymers. With cross-linking currently being investigated as a powerful strategy to increase the stability of organic semiconductor devices, novel organic azido cross-linkers might become crucial in the commercial breakthrough of organic semiconductors.

## List of Abbreviations

(HxN_3_)_2_-SiPcbis(6-azidohexanoate)silicon phthalocyanine1,3-BDSA1,3-benzenedisulfonyl azideAAPMA4-(acyl azido) phenylmethacrylateABAMPAazido ester 3-azido-2,2-bis(azidomethyl)propyl 2-azidoacetateBABAMP1,3-bis (azido acetoxy)-2,2-bis(azidomethyl) propaneBABP4,4′-bis(azidomethyl)-1,1′-biphenylBCBbenzocyclobuteneBDAPbis(2,3-diazidopropylene glycol)BHJbulk heterojunctionbu-NENAbutyl nitrato ethyl nitramineButBAMP2,2-bis(azidomethyl)propane-1,3-diyl dibutyrateCA1,3-dipolar cycloadditionCuIcopper(I) iodideDADFH1,7-diazido-*N*,*N*,*N*′,*N*′-tetrafluoro-4,4-heptanediamineDAZH1,6-diazidohexaneDEGBAAdiethylene glycol bis(azidoacetate)DFTdensity functional theoryDIANP1,5-diazido-3-nitrazapentaneDPP-10C_5_DEdiketopyrrolopyrrole with diethylpentanediyl linkerDPPTPTA3,6-bis(5-(4,4″-bis(3-azidopropyl)-(1,1′:3′,1″-terphenyl)-5′-yl)thiophen-2-yl)-2,5-bis(2-ethylhexyl)-2,5-dihydropyrrolo(3,4-c)pyrrole-1,4-dioneDUVdeep UVEGBAAethylene glycol bis(azidoacetate)ETPEenergetic thermoplastic elastomersF8BTpoly(9,9-dioctylfluorene-alt-benzothiadiazole)FPAbis(4-azido-2,3,5,6-tetrafluoro-benzoate)GAPglycidyl azide polymeriPPisotactic polypropyleneLEDlight-emitting diodeN_3_-(CPDT(FBTTh_2_)_2_)4-4′-bis(1-azido)undecane)dicyclopenta-(2,1-b:3,4-b′)dithio-phene-bis(5-fluoro-7-(5′-hexyl-(2,2′-bithiophene)-5-yl)benzo-(c)(1,2,5)thiadiazole)NIPAM*N*-isopropylacrylamideNPnanoparticleOFETorganic field-effect transistorOLEDorganic light-emitting diodeOPVorganic photovoltaicsOSCorganic solar cellP3HTpoly(3-hexylthio-phene-2,5-diyl)PAphosphoric acidPC_61_BMphenyl-C_61_-butyric acid methyl esterPC_71_BM(6,6)-phenyl-C_71_-butyric acid methyl esterPCBMphenyl-C_n_-butyric acid methyl ester (in general C_61_)PCEpower conversion efficiencyPDMSpoly(dimethylsiloxane)PEDTpoly(3,4-ethylenedioxythiophene)PEMFCsproton exchange membrane fuel cellsDMFCsdirect methanol fuel cellsPETKAApentaerythritol tetrakis(azidoacetate)PFBApropane-1,3-diyl bis(4-azido-2,3,5,6-tetrafluorobenzoate)PMIPKpoly(methyl isopropenyl ketonePMMApoly(methyl methacrylatepoly-AMMOpoly(3-azidomethyl-3-methyloxetane)poly-BAMOpoly(3,3–bis(azidomethyl)oxetane)POSSpolyhedral oligomeric silsesquioxanespppolypropylenePSSHpoly(styrenesulfonic acid)PTAApoly(triaryl amine)PTPApoly(triphenylamine)PVK:PBDpoly(vinylcarbazole)-2-tert-butylphenyl-5-biphenyl-1,3,4-oxadiazolrGOreduced grapheneSDO 4,4′-oxybis-benzenesulfonyl azidesFPAsterically hindered bis(fluorophenyl azides)SiIDT-BT/PC_71_BMsilaindacenodithiophene-benzotriazoleSPESpoly(ether sulfone)TAP-Actriazido pentaerythrite acetateTBA-X1,2-bis((4-(azidomethyl)phenethyl)thio)ethaneTFBpoly(9,9 di-n-octylfluorene-alt-(1,4-phenylene-((4-sec-butylphenyl)imino)-1,4-phenylene)THFtetrahydrofuranTMETA1,1,1-tris(azidomethyl)ethaneTMNTAAtrimethylol nitromethane tris(azidoacetate)TPBA1,3,5,7-tetrakis-(*p*-azidobenzyl)-adamantaneTPEtetraphenyletheneTPT-N_3_tris(4-(5′-(3-azidopropyl)-2,2′-bithiophen5-yl)phenyl)aminePBIpolybenzimidazoleX-PTCAzidecrosslinked poly triarylamine-carbazoyl azide (poly 4-(9-(6-azidohexyl)-9*H*-carbazol-3-yl)-*N*-(4-butylphenyl)-*N*-phenylaniline)

## Figures and Tables

**Table 1:** Performance of organic photovoltaic (OPV) devices where active layer crosslinking with organic azides was employed. PCE: power conversion efficiency.

Azide Crosslinker	Active Layer (1:x(CL):1)	Crosslinking Method	Reference PCE w/o CL	PCE (%) Post Annealing	PCE (%) Retention after Ageing (t, T)
**P3HT-N5**	**P3HT-N5**:0:**PC_61_BM**	DUV (60 min) ^a^	2.2	1.5	67 (40 h, 110 °C)
**N_3_-CPDT(FBTTh_2_)_2_** [62]	**P3HT**:0.2:**PC_61_BM**	thermally (130–150 °C) via CA	-	3.5	>99 (24 h, 150 °C)
**BAPB** [65]	**P3HT**:0.05:**PC_61_BM**	thermally (150 °C) via CA	3.3	3.0	92 (122 h, 150 °C) 90 (120 d, 85 °C)
**DAZH** [60]	**SiIDTBT**:0.1:**C_70_BM**	DUV (10 min)	6.0	5.7	82 (130 h, 85 °C, N_2_ atmosphere)
**TBA-X** [64]	**P3HT**:0.07:**PC_61_BM**	thermally (150 °C) via CA	3.7	3.3	87 (200 h, 120 °C)
**sFPA** [59] **^b^**	**P3HT**:0.12:**PC_61_BM**	DUV (5 min, 90 °C)	3.3	3.0	-
**Bis(BFPA)** [66] **^c^**	**P3HT**:0.1:**PC_61_BM**	DUV (5 min, rt)	-	3.4	88 (20 d in air, rt)
**DPPTPTA** [63]	**PTB7Th**:0.05:**PC_61_BM**	thermally (10 min, 150 °C)	6.7	7.3	55 (18 h, 150 °C)
**(HxN_3_)_2_-SiPc** [67]	**P3HT**:0.08:**PC_61_BM**	thermally (10 min, 80 °C)	3.4 ^d^	3.3	97 (23 h, 150 °C)
**TBT-N_3_** [61]	**P3HT**:0.05:**PC_61_BM**	thermally (20 min, 150 °C)	3.4 ^e^	3.4	60 (24 h, 150 °C)

CA = 1,3-dipolar cycloaddition; DUV = deep UV; CL = cross-linker; ^a^ Ambient fabrication; ^b^ Structured bilayer; ^c^ Used to crosslink an electron transport layer in an inverted device; ^d^ annealed at 80 °C for 10 min^; e^ annealed at 150 °C for 20 min.
